# Detrimental influences of intraluminally-administered sclerotic agents on surrounding tissues and peripheral nerves: An experimental study

**DOI:** 10.3109/2000656X.2012.675881

**Published:** 2012-06-11

**Authors:** Masahide Fujiki, Masakazu Kurita, Mine Ozaki, Hayato Kawakami, Nobuyuki Kaji, Akihiko Takushima, Kiyonori Harii

**Affiliations:** 1 Department of Plastic Surgery, Mitaka, Tokyo, Japan; 2 Department of Anatomy, Kyorin University School of Medicine, Mitaka, Tokyo, Japan

**Keywords:** plastic surgery, nerves

## Abstract

The minimally-invasive nature of sclerotherapy makes it one of the first treatment options for venous malformations, although treatment-related complications, such as peripheral nerve paralysis, have been reported in some clinical cases. However, no studies of the aetiology of the detrimental effects of intraluminally-administered sclerotic agents on the surrounding tissues, including the peripheral nerves, have yet been published. This study therefore investigated the influences of intraluminally-administered sclerotic agents on the tissues surrounding the injection site using a newly-developed rat femoral vein model. Using this model, the effects of absolute ethanol, 5% ethanolamine oleate, and 1% polidocanol were compared histologically with those of normal saline controls. Fluorescein isothiocyanate-conjugated agents were administered and the leakage of sclerotic agents through the venous wall was evaluated by fluorescence microscopy. Damage to the adjacent femoral nerve was quantitatively evaluated by counting the numbers of axons in cross-sections. All the sclerotic agents caused vascular wall injuries and leakage into the surrounding tissues. The number of axons in the femoral nerve was significantly reduced following administration of absolute ethanol or 5% ethanolamine oleate, compared with normal saline. The results of this study suggest that sclerotic agents commonly leak out the vascular lumen, and some agents can cause adjacent nerve injury. It is important to be aware of this type of complication of sclerotherapy for venous malformations when selecting appropriate therapeutic interventions.

## Introduction

Venous malformations (VM) are benign but voluminous lesions, mainly consisting of abnormally enlarged veins. The veins enlarge gradually without spontaneous regression, causing various problems, such as aesthetic disturbances, skeletal morbidities, or low-grade intravascular coagulopathy [1,2]. Localised VM can usually be treated by surgical excision, but complete surgical removal is often difficult without damaging the surrounding nerves, vessels and musculoskeletal structures and leaving conspicuous scars, and deformities. In the case of markedly enlarged VM, surgical excision is difficult because of the risk of massive or uncontrollable haemorrhaging.

Percutaneous sclerotherapy (that is, the injection of sclerotic agents via direct puncture of the lesion) has recently become the preferred therapeutic option for treating VM, because of its effectiveness and low invasiveness [3–5]. Sclerotic agents damage venous endothelial cells in VM, leading to obliteration of the vessels and reduction in the size of the lesions [6,7]. Ideally, the administered agents should be retained inside the lumens of the veins in the VM, and should not damage the surrounding healthy tissues. However, percutaneous sclerother-apy can sometimes cause serious functional and cosmetic sequelae, such as peripheral nerve disturbances [3,8–11], cutaneous necrosis [10,12] and detrimental effects on the muscle [13]. Of these, peripheral nerve disturbances, such as facial nerve paralysis or recurrent laryngeal nerve paralysis resulting in vocal cord paralysis, represent serious problems [9]. Elucidation of the mechanisms responsible for this damage to extravascular tissues is important, in order to prevent these sclerotherapy-associated complications. However, to the best of our knowledge, no information is currently available regarding the mechanisms responsible for these complications occurring after the instillation of sclerotic agents into the vascular lumen.

We hypothesised that, during VM treatment, the injected, sclerotic agents pass through the venous wall and leak out from the lesion, resulting in damage to the surrounding tissues, including the peripheral nerves. We aimed to evaluate the effects of leakage of these agents through the venous walls in VM. However, no appropriate animal model of VM has previously been reported, and we therefore developed a new animal model using the femoral veins of rats. This model met two necessary criteria: first, the site of sclerotic agent administration was not surgically dissected from the surrounding tissues; and, secondly, the venous flow at the administration site was reduced to mimic the slow vascular flow found in VM lesions [3,13].

Using this novel rat model, we studied the histopathological effects of intravenously administered sclerotic agents on the venous endothelium and surrounding tissues, with particular emphasis on damage to the adjacent femoral nerves.

## Materials and methods

### Animal model

A total of 61 Wistar rats weighing 300–350 g were used. All the operative procedures were performed under general anaesthesia with intraperitoneal administration of 50 mg/kg pentobarbital sodium. The study was designed in accordance with the Laboratory Animal Guidelines of Kyorin University School of Medicine. Seven animals died intraoperatively or postoperatively and were therefore excluded from the analysis.

**Figure 1:**
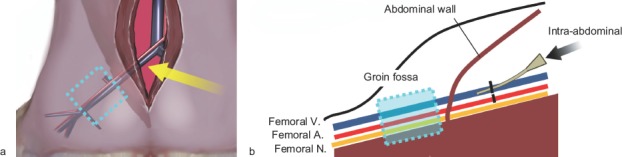
Schema of the experimental animal model. (a) The external iliac vessels were approached via a mid-abdominal incision (yellow arrow) and initially ligated using 4–0 silk sutures. The section of the femoral vein distal to the inguinal ligament was regarded as a model of slow blood flow vascular lesions (venous malformation) without surgical invasion. (b) Under direct observation of the external iliac vein, 0.5 ml of each sclerotic agent was injected retrogradely into the femoral vein from the external iliac vein distal to the ligature point (black arrow).

Using an intraperitoneal approach, the rat external iliac vein was dissected and ligated proximally, resulting in accumulation of its intraluminal venous flow in the groin fossa. The external iliac artery was also ligated to reduce back flow from the femoral vein. Dissection of the femoral vein at the groin fossa was avoided to allow investigation of the influences of sclerotic agents on the adjacent intact tissues, including the femoral nerve. Sclerotic agents were administered via a vascular catheter punctuated at the external iliac vein (Figure 1). The study was designed in accordance with the Laboratory Animal Guidelines of Kyorin University School of Medicine and the protocol was approved by the local animal experiment committee.

### Sclerotic agents

Four kinds of sclerotic agents routinely used for the treatment of VM in our institution were used: an optimised dose of 0.5 ml of absolute ethanol (ET), 5% ethanolamine oleate (EO) (composed of a 1:1 mixture of 10% EO [Oldamin®, Fuji Chemical Industry Co., Toyama, Japan] and pure water) and 1% polidocanol (PL) (Aetoxysclerol®, Sakai Chemical Industry, Co., Osaka, Japan) [3,10,13]. Normal saline (NS) was used as a control.

### Assessment of acute-phase alterations by direct observation of the dissected vein

**Figure 2:**
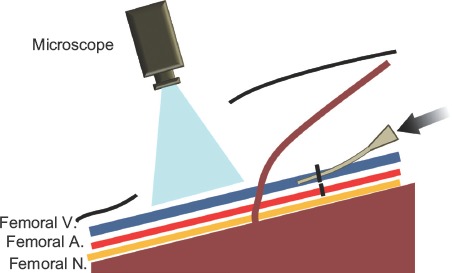
Schema of the animal model under direct observation. To assess the acute-phase alterations that occurred after the administration of the sclerotic agents, each agent was injected through the external iliac vessels under direct microscopic observation of the groin fossa. V = vein; A = artery; N = nerve.

To investigate acute-phase phenomena, the sclerotic agents were injected as described above, after dissection of the groin fossa. Any subsequent changes were observed for 15 minutes using an operating microscope (OPMI 6CH/S3, Carl Zeiss Meditec, Japan) and recorded using an integrated digital video camera after injection of ET, EO, PL, or NS (Figure 2).

### Histological assessment

The femoral vein and surrounding tissues were removed en-bloc at 24 hours and at 7 days after the injection of ET, EO, PL, or NS (*n* = 2 for each sclerotic agent and time point), fixed with 10% formalin, dehydrated and embedded in paraffin. Each sample was then sectioned axially (0.4 μm thickness) and stained with haematoxylin-eosin and Elastica van Gieson.

### Transmission electron microscopy (TEM)

Eight tissue samples (*n* = 2 for each sclerotic agent) were taken 7 days after injection, sectioned and fixed with 2% glutaraldehyde. The femoral vein from each sample was then post-fixed with 1 % osmium tetroxide, dehydrated in alcohol and embedded in epoxy resin. Ultra-thin sections (90 μm thick) were stained with uranyl acetate and lead citrate to highlight the venous endothelium and were observed by TEM (JEM-1010 transmission electron microscope, JEOL, Tokyo, Japan).

### Assessment of vascular leakage of sclerotic agents using fluorescein isothiocyanate (FITC)

To detect leakage of sclerotic agents through the venous wall, a mixture of FITC-conjugated-sclerotic and non-conjugated-sclerotic agent was injected into the femoral vein, as described above (*n* = 2 for each mixture, total of eight samples). The injection mixture consisted of the sclerotic agent or saline plus 10 mg/ml FITC (Sigma-Aldrich Corporation, St. Louis, MO) dissolved in acetone (as a solvent), at a ratio of 10:1 (final FITC concentration 1 mg/ml). Five minutes after injection, the samples were immediately frozen *in situ* in liquid nitrogen. The samples were then embedded in OCT compound and rapidly frozen in liquid nitrogen, before being cut using a cryostat and observed under a fluorescence microscope (Inverted Microscope IX71, Olympus, Tokyo, Japan).

### Assessment of neural damage

Neural damage was quantitatively analysed by counting the number of axons in cross-sections of the femoral nerve, located adjacent to the femoral vein. Seven days after injection of the sclerotic agents (*n* = 4 for each sclerotic agent, total of 16 samples), a portion of the right femoral nerve just distal to the dominant branch of the quadriceps femoris muscle was obtained using an operating microscope and fixed with 2% glutaraldehyde. An equivalent portion of the left, non-injected femoral nerve was also obtained and fixed. The nerves were then fixed with 1% osmium tetroxide, dehydrated in alcohol and embedded in epoxy resin. Semi-thin (1 mm thick) sections were cut and stained with 1% toluidine blue for examination by light microscopy. The numbers of axons in the right and left femoral nerves had previously been confirmed to be almost identical. The ratio of the total number of axons (R_axon_) in the right (injected) nerve to that in the left (untreated) nerve was calculated for each animal. The experimental protocol is summarised in Table I.

**Table I:** Summary of experimental protocol.

Timing (observation or sampling)	Assessment	Number of animals (sclerotic agents) used
During injection	Direct vision	2 (ET/EO/PL)
5 minutes after injection	FITC	2 (ET/EO/PL/NS)
24 hours after injection	HE & EVG	2 (ET/EO/PL/NS)
7 days after injection	HE & EVG	2 (ET/EO/PL/NS)
	TEM	2 (ET/EO/PL/NS)
	Neural damage	4 (ET/EO/PL/NS)

FITC = fluorescein isothiocyanate; HE = haematoxylin and eosin; EVG = Elastica van Gieson; TEM = transmission electron microscopy; ET = ethanol; EO = ethanolamine oleate; PL = polidocanol; NS = normal saline.

### Statistical analysis

Differences in R_axon_ between samples treated with the different sclerotic agents and normal saline-treated samples were analysed using one-way analysis of variance to test for significant differences between age groups. Multiple comparison analysis was performed using Dunnett's test. Values of *p* < 0.05 were considered to be statistically significant. Data are presented as the mean ± standard deviation.

## Results

### Acute–phase changes noted by direct observation

**Figure 3:**
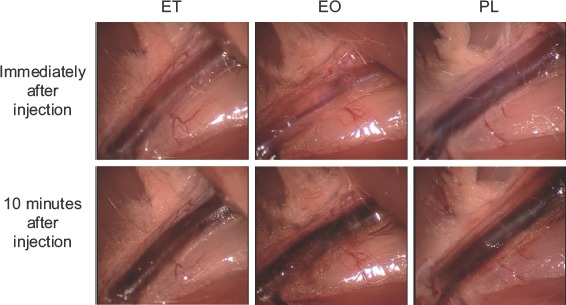
Findings immediately and 10 minutes after administration of sclerotic agents.

Femoral veins injected with each sclerotic agent showed specific alterations visible through the transparent vein, by direct observation. After injection of ET, small intravenous thrombotic particles immediately formed, followed by further thrombotic changes, including gradual discolouration of the vein. In contrast, no instantaneous thrombotic changes occurred after injection of EO or PL, although thrombotic discolouration began several minutes later and the perivascular connective tissues gradually became stained a blood-like colour, possibly as a result of the exudation of blood cells or reactive dilatation of the capillary vessels (Figure 3).

### Histologic changes in the venous endothelium and surrounding tissue

Several histologic changes could be observed after injection of the sclerotic agents. These included various degrees of dilatation of the vein, thrombus formation, extravascular infiltration of inflammatory cells, and exfoliation of the endomembrane. Twenty-four hours after the injection of ET, the thrombus had filled the venous lumen and the vein was dilated accordingly, while no such alterations occurred following the injection of NS. Although the endomembrane was frequently exfoliated, the extravascular infiltration of nucleated cells was less evident. These changes degraded the sections obtained at 7 days after injection.

**Figure 4:**
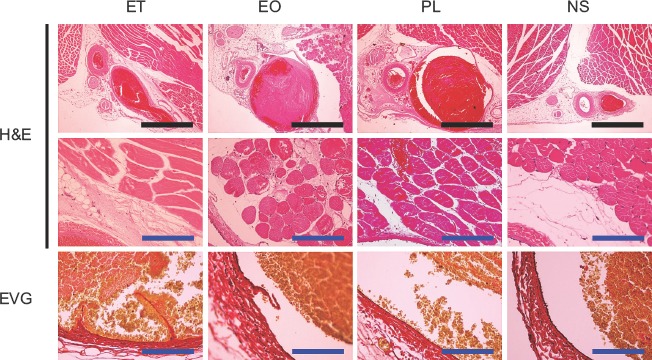
Representative histological findings of the femoral vein and surrounding tissue 24 hours after injection of sclerotic agents. The black and blue scale bars represent 100 mm and 10 mm, respectively. H&E = haematoxylin and eosin; EVG = Elastica van Gieson.

**Figure 5:**
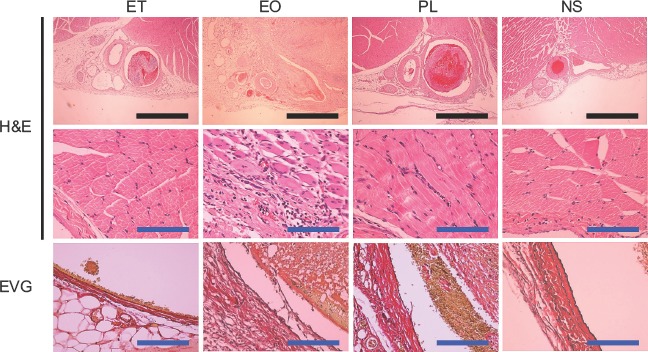
Representative histological findings of the femoral vein and the surrounding tissue 7 days after the injection of sclerotic agents. The black and blue scale bars represent 100 mm and 10 mm, respectively. H&E = haematoxylin and eosin; EVG = Elastica van Gieson.

Samples taken 24 hours after the injection of EO and PL demonstrated more obvious vascular dilatation than samples taken after the injection of ET. The EO and PL samples showed infiltration of red blood cells and inflammatory cells into the tissue surrounding the veins. Exfoliation of the endomembrane was frequently observed in EO, but not PL samples. Extravas-cular inflammation and exfoliation of the endomembrane were more marked in the EO samples obtained on the 7th day. Although exfoliation of the endomembrane was confirmed in the PL samples obtained on the 7th day, less destruction was seen than in the ET and EO samples. Representative findings and an evaluation of the histologic changes observed are shown in Figures 4 and 5.

### Detrimental effects of sclerotic agents on the endothelium observed by TEM

**Figure 6:**
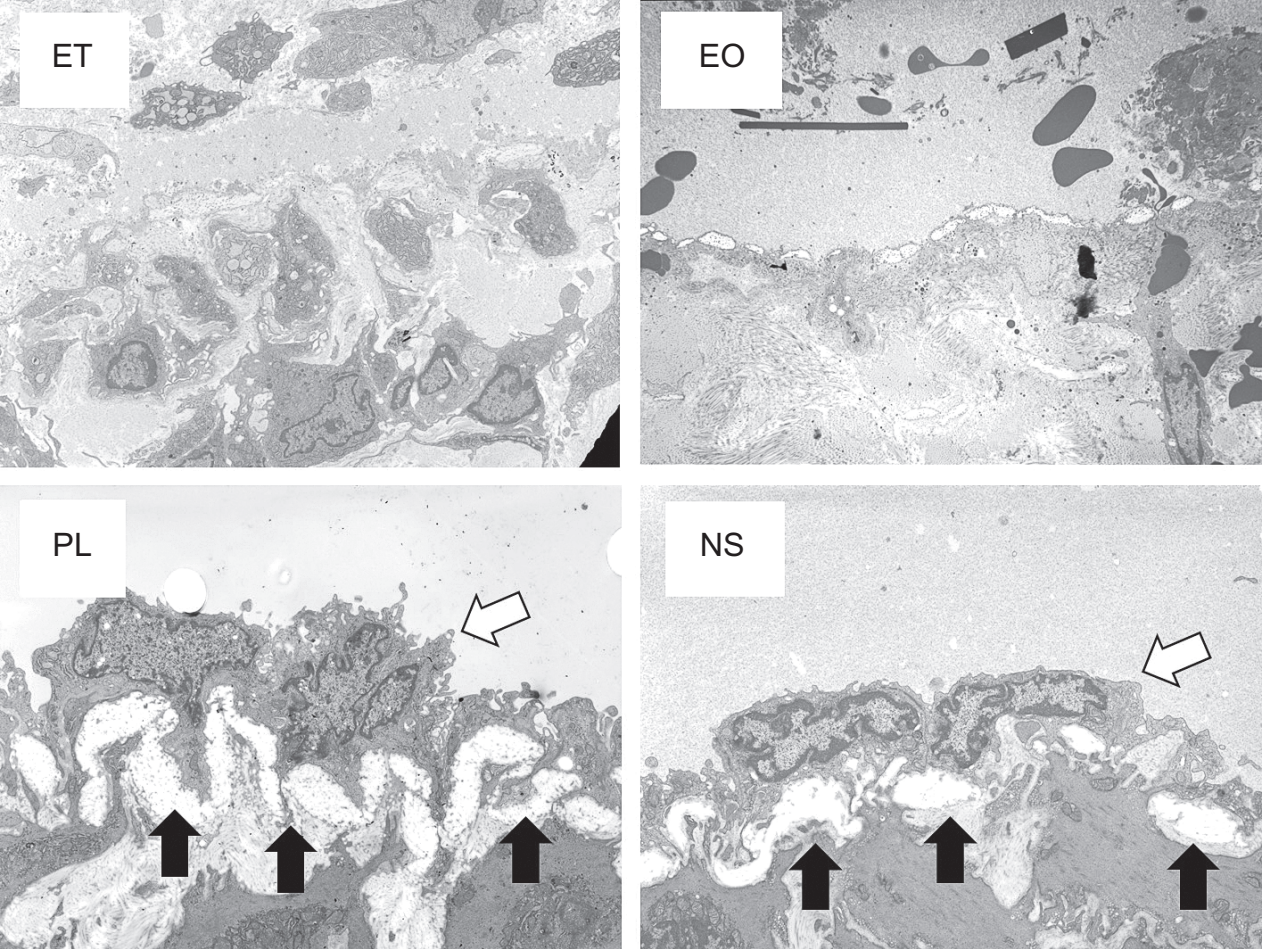
TEM findings of the femoral vein endothelium 7 days after the injection of sclerotic agents. The white arrows indicate endothelial cells and the black arrows indicate the endothelial basement membrane.

TEM showed that the endothelial cells and structures were preserved at 7 days after the injection of NS and PL and the endothelial basement membrane had thickened. In contrast, these endothelial structures had been destroyed in the EO and ET samples (Figure 6).

### Extravasation of sclerotic agents

**Figure 7:**
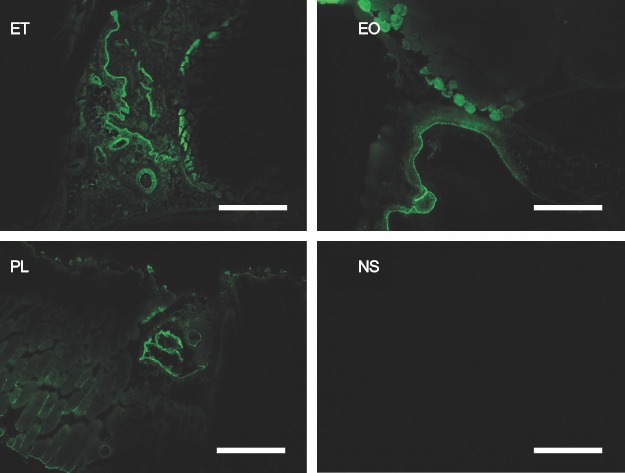
Fluorescence microscopical findings 5 minutes after the injection of a mixture of sclerotic agents and FITC. The ET, EO, and PL samples were stained with conjugated FITC. No fluorescent signals were detected in the NS samples. Scale bars represent 500 mm. FITC = fluorescein isothiocyanate; ET = ethanol; EO = ethanolamine oleate; PL = polidocanol; NS = normal saline.

The venous wall and perivascular tissue were stained by FITC in all the specimens that were cryofixed at 5 minutes after the injection of a mixture of sclerotic agents and FITC, indicating that the sclerotic agents remained in the vascular lumen, but had also leaked into the surrounding perivascular tissues through the venous wall. No FITC staining was seen in the specimens treated with normal saline and FITC (Figure 7).

### Effects of sclerotic agents on the number of axons in the femoral nerve

**Figure 8:**
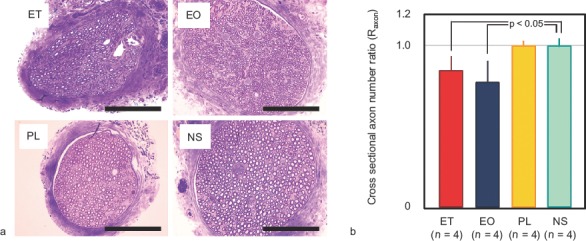
Axon numbers in cross-sections of the femoral nerve. (*a*) Cross-section of the femoral nerve (toluidine blue staining). Scale bars represent 10μm. (*b*) Ratio of the cross-sectional axon number on the treated side to that on the untreated side (R_axon_). ET and EO significantly reduced the axon number compared with the NS control (*p* < 0.05). Error bars indicate standard deviation. ET = ethanol; EO = ethanolamine oleate; PL = polidocanol; NS = normal saline.

The R_axon_ values in the ET-, EO-, PL- and NS-injected animals were calculated as 0.84 ± 0.09, 0.78 ± 0.13, 1.00 ± 0.03, and 1.00 ± 0.05, respectively. ET and EO significantly reduced cross-sectional axon numbers, in comparison with the NS control (*p* < 0.05). Representative histologic findings and axon numbers are shown in Figure 8.

## Discussion

Although several clinical studies have reported the occurrence of nerve paralysis after sclerotherapy for VM [3,8,9], the effects of intravenously-administered sclerotic agents on the surrounding tissues and especially on the adjacent nerves has not previously been reported, to the best of our knowledge. Given the lack of a suitable animal model of VM, we developed a new model using normal rat femoral veins at the groin fossa and used this model to evaluate the effects of intraluminally-administered sclerotic agents on the surrounding healthy tissues, including the peripheral nerves. This VM model enabled the effects of intravenously administered sclerotic agents to be evaluated not only on the veins, but also on the surrounding perivascular tissue, including the adjacent peripheral nerves.

In this study, the administration of ET resulted in the immediate formation of small thrombotic particles, followed by the destruction of endothelial structures. The current histologic findings, as well as those of previous studies [6,14], suggested that the detrimental effect of ET resulted from strong and rapid thrombosis formation and endothelial injury. The changes peaked during the early phase of drug administration, without prominent inflammation.

In contrast, the administration of EO or PL caused no such sudden thrombosis formation, although the injected vein had become dilated and filled with large thrombi in histologic sections obtained 24 hours after injection. The frequency of exfoliation of the endomembrane was similar or even greater in 7-day samples, compared with 24-h samples. This process was accompanied by marked inflammation. A previous *in vitro* study indicated that endothelial injury induced by surface-activating agents such as EO and PL caused increased surface expression of several cell-adhesion molecules, such as P-selectin and E-selectin [14], thus promoting the adhesion and activation of leukocytes. Inflammation and thrombosis formations were amplified by these molecular processes [15,16]. Our results were in accordance with those of previous studies and the degree of histologic alteration was much greater in EO samples compared with PL samples.

We performed several other experiments to investigate the effects of sclerotic agents on the surrounding tissue, especially the adjacent nerves. Intraluminal retention of the administered agents and infiltrative leakage into the surrounding perivascular tissues were clearly demonstrated by administration of a mixture of sclerotic agents and FITC. Although objective quantitative comparisons of the effects among the different sclerotic agents were difficult, our results demonstrated the detrimental effects of sclerotic agents on the perivascular tissues, even after careful administration. Our results also showed a reduction in the number of axons in the femoral nerve after ET or EO injection. This reduction is thought to be the result of proximal nerve injury and subsequent Wallerian degeneration [17], possibly caused by the infiltration of sclerotic agents.

The most significant limitation of this study was the fact that some aspects of our model differed from a true VM. In particular, the histologic structure of the vascular wall was different from that seen in VM. VM usually shows a significant lack of involvement of the normal surrounding smooth muscle and elastic tissues, resulting in vessels with a larger than normal luminal diameter, lined with a single endothelial layer and with scarce supportive tissue [18,19]. The relative toughness of the normal veins in our model may therefore have caused an underestimation of the destructive influences of the sclerotic agents on the surrounding tissue. Although EO demonstrated a detrimental effect on the nerves, PL failed to show such an effect, even though both agents are classified as surface-activating agents. This discrepancy may have resulted from intrinsic differences between their sclerotic activities. However, it is important to note that the failure to detect a negative effect of PL on the femoral nerve in our study does not necessarily guarantee its safety in the clinical setting.

Clinically, the kinetics of vascular anomalies vary widely in complexity, even within morbidities classified as VM; that is, some cases show higher vascular flow together with an arteriovenous shunt of unknown cause [3]. In such cases, the neural damage may be caused by other mechanisms, such as direct ischaemia or congestion of the dominant vessels of the peripheral nerves. The situation is more complex in other vascular morbidities, such as arteriovenous malformations, and caution should therefore be exercised when interpreting the results of the current study and applying them to clinical practice. Nonetheless, the fact that several types of sclerotic agents can inherently damage the surrounding perivascular tissues, including the nerves, is an important observation, suggesting that considerable care is necessary to avoid neural damage in clinical applications.

In conclusion, our model demonstrated that some types of sclerotic agents (such as ET and EO) leak out through the vascular wall after injection and can have subsequent detrimental influences on the adjacent peripheral nerves. In light of the fact that even accurate administration of sclerotic agents may cause complications, surgical excision or low-dose repetitive sclerotherapy should be considered in cases where the VM lesion is located close to the constitutive peripheral nerves.
